# Marine endophytes: biosynthetic engines for novel bioactive metabolites

**DOI:** 10.3389/fmicb.2025.1684777

**Published:** 2025-10-08

**Authors:** Cun-Cui Kong, Jia-Yi Wang, Bo-Hao Shan, Hong-Xia Zhang, Song Qin, Cheng-Gang Ren

**Affiliations:** ^1^College of Horticulture, Ludong University, Yantai, Shandong Province, China; ^2^Key Laboratory of Coastal Biology and Biological Resources Utilization, Yantai Institute of Coastal Zone Research Chinese Academy of Sciences, Yantai, China

**Keywords:** marine endophytic fungi, bioactive metabolites, strain improvement, fermentation optimization, biosynthetic potential

## Abstract

Marine endophytes are prolific sources of structurally diverse secondary metabolites with significant pharmaceutical potential, including anticancer, antimicrobial, and antioxidant agents. However, their commercial utilization is hindered by genomic instability in axenic cultures and inconsistent metabolite yields. While current studies focus on symbiotic interactions and compound discover, critical gaps persist in harnessing their biosynthetic capabilities. This review synthesizes knowledge on marine fungal metabolites and proposes a paradigm shift toward resource-driven research. It addresses strain improvement limitations and suggests strategies like mutagenesis, protoplast fusion, and metabolic engineering to bolster production stability and efficiency. The paper also discusses biological process optimization, including fermentation tuning, inducer and precursor addition, and adsorbent use, to enhance natural product synthesis. By identifying these research gaps and proposing a strategic roadmap, the review advances the stable and scalable production of bioactive metabolites, unlocking the commercial and therapeutic potential of marine endophytic fungi.

## 1 Introduction

Marine endophytes, which live within the tissues of their hosts, are ecologically significant in the oceanic ecosystem. They engage in a symbiotic relationship with their hosts, influencing growth and evolution through complex signal transduction pathways and providing protective substances that enhance the host’s survival value (Handayani et al., 2019; Rodriguez et al., 2009; Dastogeer et al., 2018). Building upon the xenohormesis hypothesis—which proposes that heterotrophs sense stress-induced chemical cues from other species to mount preemptive defenses—marine endophytes may utilize analogous mechanisms to perceive host-derived stress signals. This signaling interplay could trigger adaptive responses in endophytes, including the production of bioactive metabolites that synergistically enhance host defense (Howitz and Sinclair, 2008). Analogous to the biosynthesis of mycosporine-like amino acids (MAAs) in cyanobacteria—stress-induced molecules generated via conserved biosynthetic gene clusters that accumulate in marine consumers—metabolite induction in marine endophytes likely originates from fungal stress-responsive pathways (Jain et al., 2017). These pathways may function independently of host metabolite replication, consistent with observations that marine endophytic fungi represent a rich source of structurally unique bioactive compounds. Indeed, marine endophytic fungi have been demonstrated to be a rich source of biologically active natural products with unique structures and potent medicinal properties (Strobel and Daisy, 2003; Bugni and Ireland, 2004).

Marine endophytic fungi are known to produce a plethora of bioactive secondary metabolites, such as steroids, alkaloids, terpenoids, and peptides, many of which possess biological activities including anti-inflammatory, antioxidant, antimicrobial, and antitumor properties (Mohamed El-Bondkly et al., 2020; El-Bondkly et al., 2021; Santos et al., 2019; Tan and Zou, 2001). These include potential anticancer drugs, antimicrobial agents, antifungal compounds, antiviral substances, and more (Mm et al., 2015; El-Gendy et al., 2016; Strobel, 2018; El-Gendy et al., 2018; El-Gendy et al., 2008a; El-Gendy et al., 2000; El-Gendy et al., 2008b; El-Bondkly et al., 2012; El-Gendy and El-Bondkly, 2010; El-Gendy and El-Bondkly, 2011; El-Gendy et al., 2017). The prospect of utilizing endophytic fungi for the sustainable production of life-saving drugs is highly promising. Despite the identification of a multitude of bioactive molecules from marine endophytic fungi over the past two decades, the commercial exploitation of these organisms as a source of biologically active secondary metabolites has yet to see substantial breakthroughs. The primary constraint on the commercialization is believed to be the reduction in product yield following the subculturing of endophytic fungi under sterile conditions, which may be due to the loss of biosynthetic pathways or changes in regulatory mechanisms (Sandrawati et al., 2020; El-Bondkly and El-Gendy, 2010).

This review synthesizes literature evidence supporting the presence of host-independent biosynthetic machinery within endophytic fungi. It then explores the spectrum of marine endophytes and their secondary metabolites and highlights the need for a deeper understanding of the intricate interactions between marine endophytic fungi and their hosts. Advances in genetic engineering, such as CRISPR-Cas9 technology, offer new avenues for strain improvement, potentially enhancing the production of desired metabolites (Bary, 1866; Hardoim et al., 2015; Wilson, 1995). Furthermore, cutting-edge fermentation optimization techniques, including systems biology approaches and synthetic biology, are discussed to create an optimal culture environment for the sustainable and high-yield production of valuable secondary metabolites (Hallmann et al., 1997; Petrini, 1991; Muralikrishnan, 2013). By integrating these strategies, the review aims to provide a roadmap for harnessing the full potential of marine endophytic fungi in the biotechnological and pharmaceutical industries.

## 2 Definition, status and diversity of marine endophytic fungi

Endophytes, first identified by Bary (1866), are defined as organisms that can colonize the interior of plants without causing harm, a definition that has evolved over time (Hardoim et al., 2015; Wilson, 1995; Hallmann et al., 1997; Petrini, 1991) definition encompasses a broad range of organisms, including bacteria, fungi, mycoplasmas, and archaea (Muralikrishnan, 2013; Zhao et al., 2011; Stone et al., 2000; Hollants et al., 2011). Endophytic fungi are particularly notable for their potential to produce a diverse array of bioactive compounds. Estimates suggest that there may be over a million species of endophytic fungi, with only a fraction described (Fau et al., 1997; Dreyfuss and Chapela, 1994). These fungi are found in a variety of marine organisms, from plants to invertebrates and vertebrates (Sandrawati et al., 2020; Wu and Morris, 1973), and they play crucial roles in promoting growth, enhancing disease resistance, and improving environmental stress tolerance in their hosts (Cheng et al., 2020). The symbiotic relationship between endophytic fungi and their hosts often results in the production of secondary metabolites with potential applications in medicine, agriculture, and industry (Cheng et al., 2020; Strobel, 2002).

**Figure 1:**
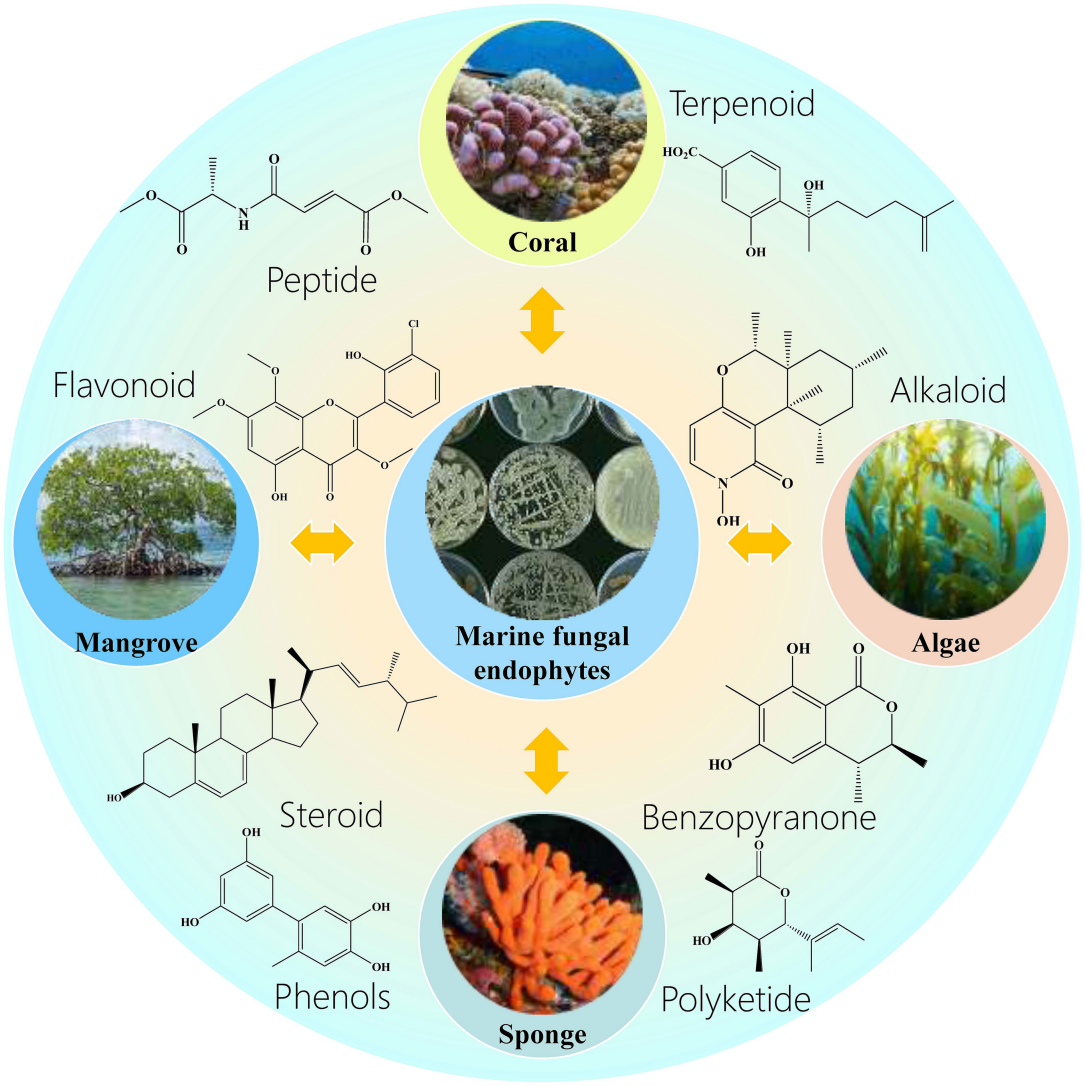
Marine endophyte host organisms and their associated bioactive compound categories. This figure systematically categorizes the major marine host organisms (Corals, Mangrove, Algae, Sponge) and their corresponding bioactive compound classes (Peptide, Terpenoid, Flavonoid, Alkaloid, Steroid, Xanthone, Benzopyranone, Polyketide) produced by symbiotic endophytic fungi.

The identification of marine endophytic fungi has been advanced by molecular methods, which overcome the limitations of traditional culture methods (El-Gendy et al., 2008a; El-Gendy et al., 2010; Song et al., 2021; Fadiji and Babalola, 2020; Higgins et al., 2007). Endophytic fungi exhibit a range of host specificities, from narrow to broad, and their composition is influenced by factors such as geography and host age (Gao et al., 2018; González-Menéndez et al., 2014). Gao et al. (2018) found that even within the same geographical location, different sponge species harbor distinct endophytic fungal communities, highlighting the specificity and diversity of these associations. Marine endophytic fungi are a rich source of bioactive compounds, with marine algae and corals being particularly prolific sources (El-Demerdash et al., 2020; Couttolenc et al., 2015). These fungi produce compounds with anticancer, antioxidant, antimicrobial, antiviral, and other properties (El-Bondkly et al., 2021; Burragoni and Jeon, 2021; Kamat et al., 2020). For instance, the endophytic algal fungus *Paecilomyces* var*iotii* produces indole derivatives with cytotoxic effects on cancer cell lines, while the red algal endophytic fungus *Microsporum* sp. produces compounds that induce apoptosis in HeLa cells. The potential of marine endophytic fungi as a source of novel bioactive compounds is vast and largely unexplored (Drake et al., 2018). Figure 1 lists marine endophyte host organisms and their associated bioactive compound categories. As research progresses, these fungi are poised to become increasingly significant in the development of new pharmaceuticals and agricultural products (El-Demerdash et al., 2020; Couttolenc et al., 2015). Their unique ecological niches, characterized by conditions such as high salinity and pressure, drive the production of specialized metabolites with potential for novel pharmaceutical applications (Sharma et al., 2020; Tidke et al., 2019).

## 3 High value compounds found in marine endophyte fungi

Marine fungal endophytes are known to establish symbiotic relationships with marine organisms such as sponges, corals, algae, and mangroves, producing a variety of bioactive metabolites with potential applications in agriculture, pharmaceuticals, food, and cosmetics (Cheng et al., 2020; Gao et al., 2018; Gouda et al., 2016). These metabolites, which include ayamycin, benzopyrone derivatives, and iso-coumarin derivatives, are often the basis for the development of drugs to treat various diseases (Cheng et al., 2020; Gao et al., 2018; Gouda et al., 2016). The secondary metabolites produced by marine endophytic fungi are diverse, encompassing alkaloids, benzopyranones, chinones, flavonoids, phenolic acids, quinones, steroids, saponins, tannins, terpenoids, tetra ketones, xanthones, and more (El-Gendy et al., 2016; Strobel, 2018; El-Gendy et al., 2008a; El-Gendy et al., 2000; El-Gendy et al., 2008b; El-Gendy et al., 2016; El-Gendy et al., 2003). These compounds are not only chemically diverse but also biologically active, making them valuable for various industries.

The symbiotic relationship between endophytic fungi and their hosts is mutually beneficial, with the fungi obtaining nutrients while also enhancing the host’s environmental adaptability through the production of secondary metabolites (Bamisile et al., 2018). For instance, fungal endophytes from algae promote algal growth and are isolated through careful culturing and surface disinfection to remove epiphytes (Wu and Morris, 1973; Teixeira et al., 2019; Zhang et al., 2016). The metabolites produced by these associations are often peptides, polyketones, lactones, alkaloids, and terpenes, which are listed in Table 1. Sponges, as primitive metazoans, have been a source of a wide array of secondary metabolites and are considered a rich source of new drug candidates (Love et al., 2009; Faulkner, 2000). The fungi within sponge tissues, which can constitute a significant portion of the biomass, are believed to be the true producers of some sponge natural products (Cheng et al., 2020; Friedrich et al., 2001). Researchers have identified a range of compounds from sponge-derived endophytic fungi, including alkaloids, terpenoids, amino acids, nucleosides, cyclic peptides, polyethers, macrolides, peroxides, polyenes, polyalkynes, and steroids, many of which exhibit antiangiogenic, antimicrobial, antiparasitic, antitumor, antiviral, hemolytic, and cytotoxic activities (Elsebai et al., 2021; Sun et al., 2017). Corals, a class of marine invertebrates, is also a significant source of medicinal value, particularly due to the metabolic products of the symbiotic microorganisms (El-Demerdash et al., 2020; Liu et al., 2019). The study of the secondary metabolites of coral-associated fungi is an important field, with recent research focusing on their roles in antitumor, antibacterial, antifouling, and osteoclast differentiation inhibition (El-Demerdash et al., 2020; Liu et al., 2019). Galkiewicz’s work marks the first report of fungi extracted from deep-sea corals, providing insight into the microbial community’s constituent members and their potential functions (Galkiewicz et al., 2012). Mangrove ecosystems, characterized by their unique saline environment and rich mineral resources, are home to a diverse array of endophytic fungi that form the second largest group of marine microorganisms (Glaser and Mayer, 2009; Xing and Guo, 2011). The endophytic fungi in mangroves, including *Aspergillus*, *Penicillium*, *Trichoderma*, *Pestalotiopsis*, and *Streptomyces*, produce a wide range of metabolites such as coumarin, chromone, terpenoids, alkaloids, peptides, quinones, and esters (Deshmukh et al., 2018; Ananda and Sridhar, 2002). These compounds represent a vast natural pharmacy with novel structures and significant biological activities (Deshmukh et al., 2018; Xin et al., 2013; Li et al., 2010).

**Table 1:** Examples of high value secondary metabolites isolated from marine endophytic fungal.

Compound name	Biological activity	Fungal source (Genus/Species)	Source host	Literature reference
Nigerasperone A-C	Inhibitory effects towards A-549 and SMMC-7721 cell lines and antifungal activity towards *C. albicans* and DPPH scavenging	*Aspergillus niger* EN-13	Brown alga *Colpomenia sinuosa*	Zhang et al. (2007) and Zhang et al. (2010)
Chrysin	Induced apoptosis, G1 phase cell cycle arrest, MMP loss and ROS production	*Chaetomium globosum*	Green alga Chaetomorpha media	Kamat et al. (2020)
Ascosalipyrrolidinone A	Antiplasmodial activity, antimicrobial and p56^lck^ tyrosine kinase inhibiting activity	*Ascochyta salicorniae*	Green alga *Ulva* sp.	Osterhage et al. (2000)
Chaetominedione	Inhibitory activity toward p56^lck^ tyrosine kinase	*Chaetomium* sp.	Alga	Abdel-Lateff (2008)
Chaetopyranin	Cytotoxic activity towardtumor cell lines	*Chaetomium globosum*	Red alga *Polysiphonia urceolata*	Wang et al. (2006)
Penicisteroids A	Antifungal and cytotoxic activities	*Penicillium chrysogenum* QEN-24S	Red alga *Laurencia* sp.	Gao et al. (2011)
Epicoccone	Antioxidative properties	*Epicoccum* sp.	Alga *Fucus vesiculosus*	Abdel-Lateff et al. (2003a)
2,3,6,8-tetrahydroxy-1-methyl-xanthone	Radical scavenging and antioxidative effects	*Wardomyces anomalus*	Alga Enteromorpha sp.	Abdel-Lateff et al. (2003b)
Isochaetoglobosin D	Antitumor activity	*Chaetomium globosum* KMITL-N0802	Marine green alga *Ulva pertusa*	Kanokmedhakul et al. (2002)
Chaetoglobosin Fex	Antitumor activity	*Chaetomium globosum* QEN-14	Green alga *Ulva pertusa*	Cui et al. (2010b)
Noduliprevenone	Inhibitor of cytochrome P450 1A activity and anticancer activities	*Nodulisporium* sp.	Alga Mediterranean sp.	Pontius et al. (2008)
Monodictysin B	Antineoplastic activity	*Monodictys putredinis*	Green alga	Krick et al. (2007)
Spartinol A	Inhibition of leukocyte elastase	*Phaeosphaeria spartinae*	Alga *Ceramium* sp.	Elsebai et al. (2009)
Citrinal A	Cytotoxic effects on the A-549 and HL-60 cell lines	*Penicillium* sp. i-1-1	Alga	Zhu et al. (2009)
Phenalenone	Cytotoxicity towards K562, SKM1 and U266 cancer cell lines	*Coniothyrium cereale*	Alga Enteromorpha sp.	Elsebai et al. (2016)
Alkaline cellulases	Applied to washing, food, chemical industry, manufacturing paper, textile and waste water treatment	*Chaetomium* sp.	Mangrove	Chinnarajan et al. (2010)
Penochalasin I	Cytotoxicity against MDA-MB-435 and SGC-7901 cells	*Penicillium chrysogenum*	Mangrove	Huang et al. (2016)
Penochalasin J	Antifungal activity	*Penicillium chrysogenum*	Mangrove	
24-hydroxylergosta-4,6,8(14),22-tetraen-3-one	Inhibited the plant pathogenic fungi	*Aspergillus clavatus*	Mangrove	Li et al. (2017)
Kotanin	Antifungal activities	*Aspergillus clavatus*	Mangrove	
(S)-5-hydroxy-2,6-dimethyl-4H-furo[3,4-g]benzopyran-4,8(6H)-dione	Inhibited Colletotrichum musae	*Aspergillus clavatus*	Mangrove	
Orlandin	Antifungal activities	*Aspergillus clavatus*	Mangrove	
Cytoglobosins C	Anticancer activities	*Penicillium* sp. V11	Mangrove	Huang et al. (2016)
Xyloketals A	Inhibiting activity against acetylcholine esterase	*Xylaria* sp.	Mangrove	Lin et al. (2001)
Isoflavone analog B	Cytotoxicity targeted to cancer cells.	*Phomopsis* sp.	Mangrove	Tao et al. (2010)
Prostaglandin analog A	Anticancer activity	*Phomopsis* sp.	Mangrove	Tao et al. (2010)
p-Terphenyl	Inhibitory towards α-glucosidase and acetyl cholinesterase	*Penicillium chermesinum*	Mangrove	Huang et al. (2011)
Phomoxanthones F	Anti-HIV activity	*Phomopsis* sp. Xy21	Mangrove *Xylocarpus granatum*	Hu et al. (2018)
Sterigmatocystin	Cytotoxic activity towards tumor cell lines	*Fungi*	Mangrove Kandelia candel	Zhu and Lin (2007)
Rubasperone A	Cytotoxicity targeted to cancer cells.	*Aspergillus tubingensis* GX1-5E	Mangrove *Pongamia pinnata*	Huang et al. (2010)
Rubasperone C	Inhibitory towards cancer cell U87MG	*Aspergillus tubingensis* GX1-5E	Mangrove *Pongamia pinnata*	Huang et al. (2010)
Sporothrins A	Inhibition activity of acetylcholine esterase	*Sporothrix* sp.	Mangrove Kandelia candel	Wen et al. (2009)
Guignardones F-I	Inhibitory activity towards *Staphylococcus aureus*and *Staphylococcus aureus*.	endophytic fungus A1	Mangrove *Scyphiphora hydrophyllacea*	Mei et al. (2012)
Eremophilane sesquiterpenes 07H239-A	Activation activity on *α*-glucosidase	*Xylaria* sp. BL321	Mangrove	Song et al. (2012)
5-epi-Asperdichrome	Antibacterial activities	*Aspergillus versicolor* HDN1009	Mangrove	Yu et al. (2018)
Versixanthones N	Cytotoxicities against five cancer cell lines (HL-60, K562, H1975, MGC803, and HO-8910)	*Aspergillus versicolor* HDN1009	Mangrove	
Versixanthones O	Cytotoxicities against cancer cell lines	*Aspergillus versicolor* HDN1009	Mangrove	
Arigsugacin I	Inhibitory activities against acetylcholinesterase	*Penicillium* sp. sk5GW1L	Mangrove Kandelia candel	Huang et al. (2013)
Terreulactone C	Inhibitory activities against acetylcholinesterase	*Penicillium* sp. sk5GW1L	Mangrove	Ding et al. (2016)
Pycnidione	Antiplasmodial, antifungal, cytotoxic activity and induced erythropoietin gene expression in human cells	*Phoma* sp.	Sponge Halichondria panacea.	Harris et al. (1993)
Microsphaeropsisin	Antimicrobial activity	*Microsphaeropsis* sp.	Sponge *Myxilla incrustans*	Rodriguez et al. (2009)
Ulocladol	Enzyme inhibitory activity	*Ulocladium botrytis*	Sponge *Callyspongia vaginalis*	Strobel (2002)
Citreonigrin B	Inhibited protein kinases	*Penicillium citreonigrum*	Sponge Pseudoceratina purpurea	El-Bondkly et al. (2021)
19-Epi-21-hydroxy-10,23-dihydro-24,25-dehydroaflavinine	Inhibitory activity towards *Bacillus subtilis*, *Staphylococcus epidermidis* and *Staphylococcus aureus* along with cytotoxicity toward HeLa, L-5178Y and PC-12 cell lines	*Aspergillus niger*	Sponge *Axinella damicornis*	Bacon (1994)
Marilines A and B	Inhibited human leukocyte elastase	*Stachylidium* sp.	Sponge Callyspongia cf. C. flammea.	Almeida et al. (2012)
Marilone A	Antiplasmodial activity	*Stachylidium* sp.	Sponge Callyspongia sp. cf. *C. flammea*	Almeida et al. (2011b)
Marilone B	Antagonistic activity towards the serotonin receptor 5-HT2B	*Stachylidium* sp.	Sponge Callyspongia sp. cf. *C. flammea*	Almeida et al. (2011b)
Glycylrubropunctatin	Anticancer and antioxidant activities	*Talaromyces verruculosus*	Sponge	Lebeau et al. (2017)
Chrysazin	Antifungal activity against *Candida albicans*	*Beauveria bassiana* TPU942	Sponge	Yamazaki et al. (2012)
Globosuxanthone A	Repressed the HCT-15 and Jurkat cells proliferation	*Beauveria bassiana* TPU942	Sponge	Yamazaki et al. (2012)
Dihydroauroglaucin	Antibacterial and antimicroalgal activity as well as inhibitory activity against tyronisase	*Eurotium chevalieri* MUT 2316	Sponge *Grantia compressa*	Bovio et al. (2019)
Flavoglaucin	Used as a broad spectrum fungicide	*Eurotium chevalieri* MUT 2316	Sponge *Grantia compressa*	Bovio et al. (2019)
Karimanone	Inhibitory activity towards *Salmonella enterica* and *Salmonella Typhi*	*Daldinia eschscholtzii*	Sponge Xestospongia sp.	Sibero et al. (2020)
Penicifurans A	Inhibitory activity against *Staphylococcus albus*	*Penicillium* sp. MWZ14-4	Sponge	Qi et al. (2013)
Penicimarins A	Antibacterial activities and cytotoxic activities	*Penicillium* sp. MWZ14-4	Sponge	Qi et al. (2013)
Sclerotiorin	Antiviral activity towards HSV and EV71	*Penicillium sclerotiorum*	Sponge	Wang et al. (2018)
Sydonol	Inhibitory activity towards *Scaphirhynchus albus* and Micrococcus tetragenus	*Aspergillus* sp.	Sponge Xestospongia testudinaria	Nukina et al. (1981)
Sydonic Acid	Antibacterial activity	*Aspergillus sydowi*	Sponge Xestospongia testudinaria	Hamasaki et al. (1978)
Hydroxysydonic acid	Antibacterial activity	*Aspergillus sydowi*	Sponge Xestospongia testudinaria	Yajima et al. (2021)
Sartorypyrone B	Inhibitory activity against three cell lines (MCF-7, NCI-H460 and A375-C5)	*Neosartorya tsunodae* KUFC 9213	Sponge Aka coralliphaga	Eamvijarn et al. (2013)
Tetrahydroaltersolanol C	Antiviral activity towards the porcine reproductive and respiratory syndrome virus	*Alternaria* sp. ZJ-2008003	Coral Sarcophyton spp.	Zheng et al. (2012)
Alterporriol P	Cytotoxic activity towards the PC-3 and HCT-116 cell lines	*Alternaria* sp. ZJ-2008003	Coral Sarcophyton spp.	Zheng et al. (2012)
Aniduquinolone AD	Antifouling activity	*Scopulariopsis* sp.	Coral G*orgonian* sp.	Shao et al. (2015)
Chrysogeamides A-E	Discriminating activity in promoting angiogenesis	*Penicillium chrysogenum*	Coral G*orgonian* sp.	Hou et al. (2019)
Asperversiamides A-C	Inhibitory activity towards *Mycobacterium Marinum*	*Aspergillus versicolor*	Coral G*orgonian* sp.	Hou et al. (2019)
Phomaethers A-B	Antibacterial activity	*Phoma* sp.	Coral G*orgonian* sp.	Shi et al. (2017)
Pestaloxazine A	Anti-EV71 activity and antiviral activities	*Pestalotiopsis* sp.	Coral Sarcophyton sp.	Jia et al. (2015)
Terreusterpenes A-B	Inhibitory activity towards BACE1	*Aspergillus terreus*	Coral Sarcophyton subviride	Qi et al. (2018)
Aszonapyrones A	Inhibitory activity against three cell lines (MCF-7, NCI-H460 and A375-C5)	*Neosartorya laciniosa* KUFC 7896	Coral *Porites lutea*	Eamvijarn et al. (2013) and Gomes et al. (2014)
Chaetomugilins	Antimicrobial, nitric oxide inhibitory, gp120-CD4-binding inhibitory, monoamine oxidase inhibitory and platelet-derived growth factor-binding inhibitory activities	*Chaetomium globosum* OUPS-T106B-6	Marine fish *Mugil cephalus*	Yamada et al. (2008)

## 4 Using endophytic fungi as production platforms for marine natural products

The necessity of employing marine endophytic fungi as production platforms for secondary metabolites is underscored by several critical factors. Firstly, the natural abundance of marine organisms that produce valuable secondary metabolites is often insufficient for large-scale pharmaceutical applications, particularly for species such as sponges and soft corals. The limited biomass of these organisms poses a significant constraint for the sustainable extraction of bioactive compounds in quantities sufficient for drug development and commercial production (El-Bondkly and El-Gendy, 2010; El-Bondkly and El-Gendy, 2012).

Marine endophytic fungi, which coexist with marine flora and fauna in a symbiotic relationship, have demonstrated the capacity to evolve unique biosynthetic pathways. This evolutionary adaptation suggests that these fungi may be the actual producers of the secondary metabolites traditionally attributed to their host organisms (Cheng et al., 2020; Gao et al., 2018). The ability of marine endophytic fungi to synthesize a range of bioactive compounds, including those with antibacterial, antifungal, and anticancer properties, positions them as promising candidates for the production platforms of these valuable metabolites (Gouda et al., 2016; Hyde, 2019).

Numerous valuable compounds have been isolated from endophytic fungi associated with marine organisms. These include antibacterial agents such as benzopyrone and isocoumarin derivatives (El-Gendy et al., 2008a; El-Gendy et al., 2000; El-Gendy et al., 2008b; El-Gendy and El-Bondkly, 2010), antifungal compounds like mycopane and sardamycin (El-Bondkly et al., 2012; El-Gendy and El-Bondkly, 2010), and anticancer agents including lovastatin (Nurunnabi et al., 2020; Sopalun and Iamtham, 2020). Additionally, bioactive compounds such as antifungal and cytotoxic polyoxygenated steroids (Penicisteroids A and B), anthraquinone, cyclopentanone, and naphthoquinone derivatives have been isolated from algae endophytes (Gao et al., 2018). Furthermore, isobenzofuranone derivatives, marilones A-C, stachylines A-D, and marilines A-C with antioxidant properties have been extracted from algicolous fungi and sponge-derived fungi (Almeida et al., 2011a; Almeida et al., 2011b; Kamat et al., 2020).

Despite the potential of endophytic fungi to produce high-value pharmaceuticals, commercial production of these fungi for drug synthesis has not yet been realized. The primary obstacle to commercialization is the reduction in target product yield following the subculturing of endophytes, which some researchers attribute to the loss of biosynthetic capabilities *in vitro* (Heinig et al., 2013). However, studies such as those by Yang et al., who conducted whole-genome sequencing and multiple sequence alignment of the paclitaxel-producing endophyte *Penicillium aurantiogriseum* NRRL 62431, have refuted this notion (Yang et al., 2014). Genomic analysis by Yang et al. revealed that *Penicillium aurantiogriseum* NRRL 62431 possesses evolutionarily distinct biosynthetic pathways for paclitaxel synthesis, with key enzymes (e.g., taxadiene synthase homologs) sharing <30% amino acid identity to those in Taxus hosts. This supports the capacity for autonomous production of secondary metabolites in axenic culture across multiple generations, though natural symbiotic metabolite exchange remains possible.

## 5 Strategies for enhancing secondary metabolite production in marine endophytic fungi

**Figure 2:**
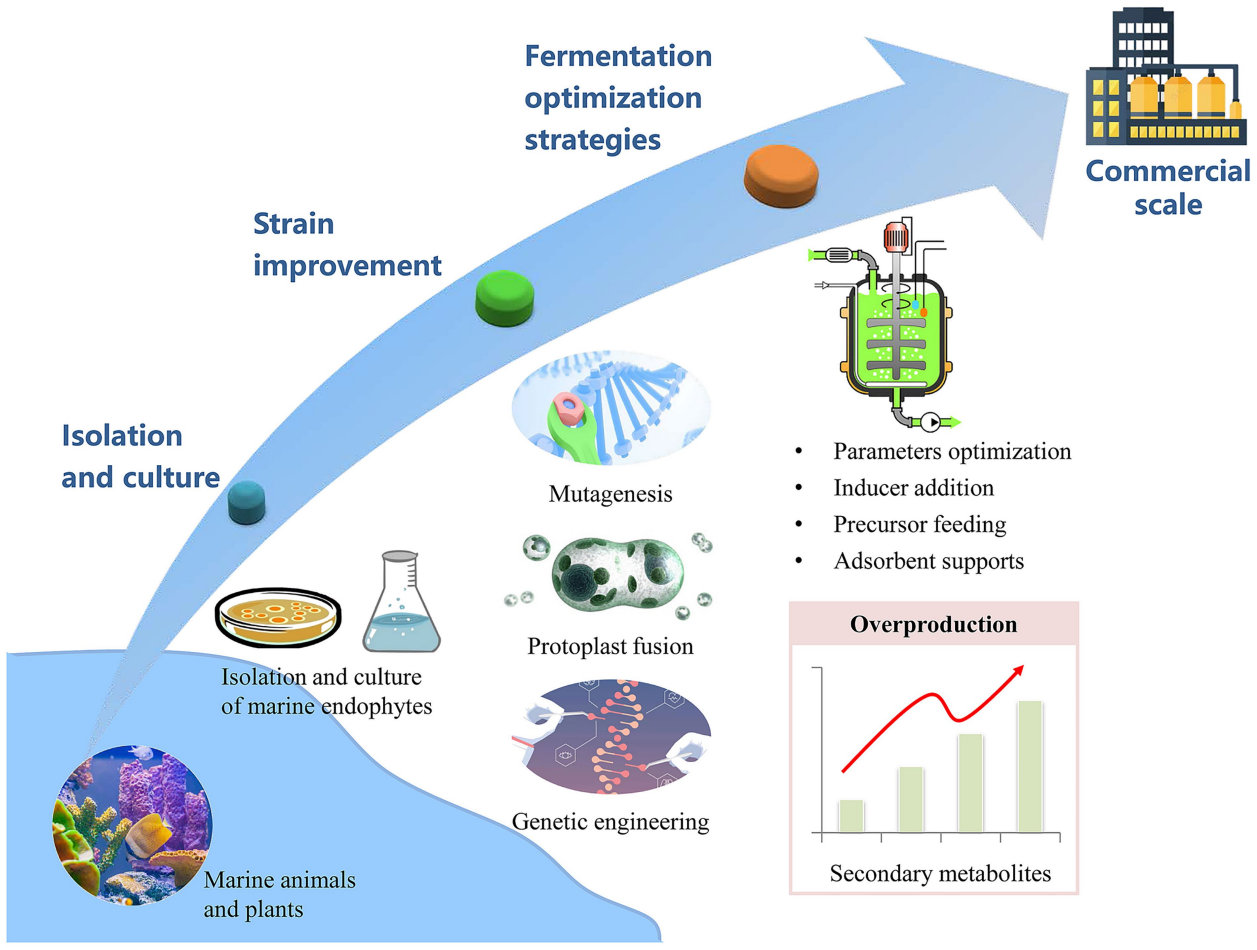
Harnessing marine endophytic fungi for industrial secondary metabolite yield.

Despite their promise as a source of natural therapeutics, marine endophytic fungi produce secondary metabolites at levels that are typically too low for commercial viability. To overcome this, strain enhancement techniques are crucial for increasing the yield and efficiency of metabolite production to a scale suitable for industrial applications. Advanced strains can then be subjected to fermentation medium optimization, which is a key to further boosting the output and productivity of these valuable compounds. Figure 2 outlines a strategic approach for the industrial-scale production of secondary metabolites derived from marine endophytic fungi.

### 5.1 Isolation and culture of marine endophytic fungi

Isolating and culturing marine endophytic fungi is essential for harnessing their secondary metabolites. It involves extracting these fungi from a variety of marine habitats while meticulously excluding epiphytic microorganisms to ensure the purity of endophytic isolates. Selecting healthy, disease-free samples is crucial to prevent the isolation of pathogenic species and to focus on endophytes with beneficial traits (Strobel and Daisy, 2003; Strobel, 2003). To minimize contamination risks, samples should be processed promptly or kept at 4 °C in temporary storage (Strobel et al., 1996; Bacon and White, 2018).

**Figure 3:**
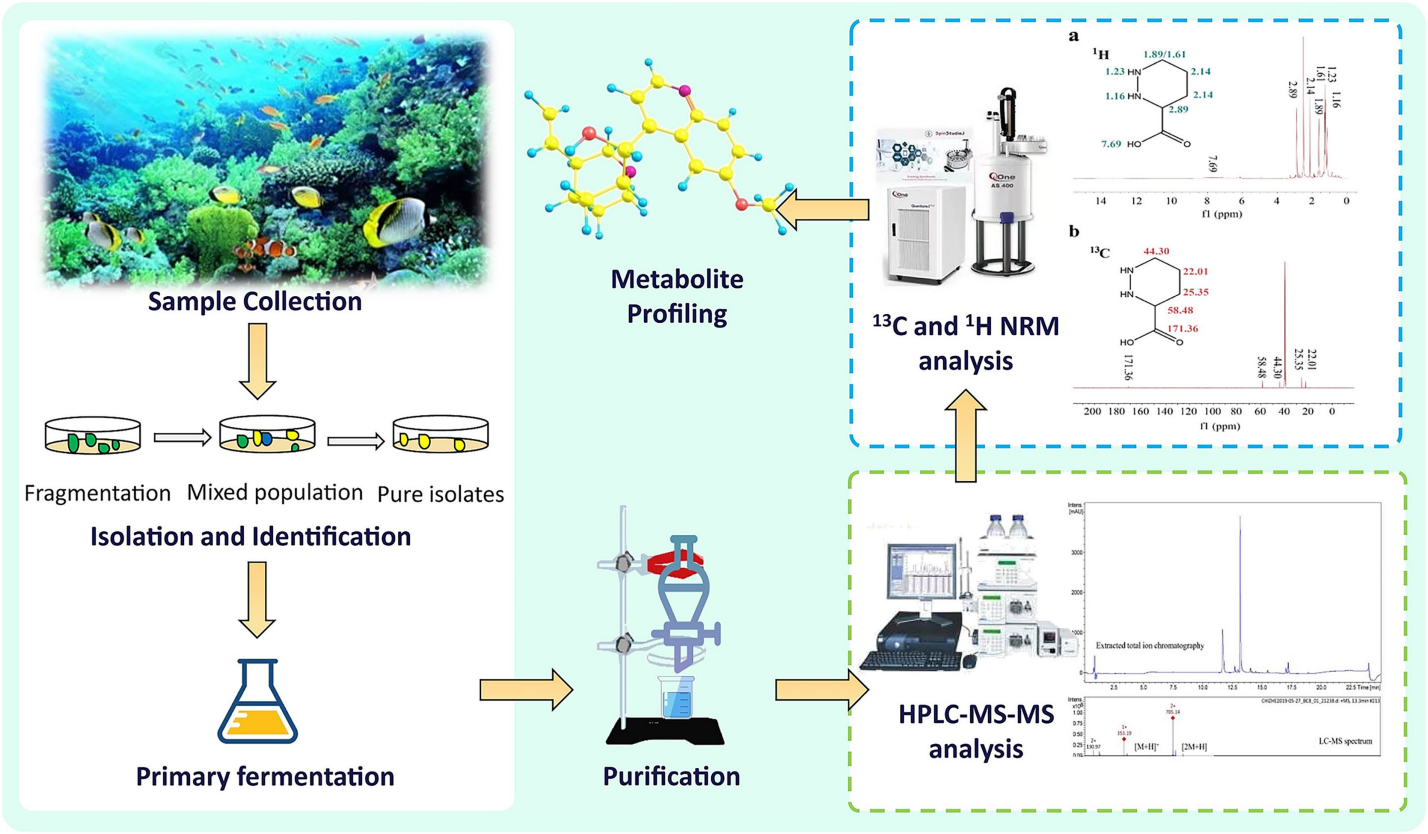
Marine endophyte isolation process and metabolite profiling.

The surface sterilization of samples, tailored to the host’s species and tissue type, is a critical step to guarantee the isolation of true endophytes (Bissegger and Sieber, 1994). This process commonly employs mechanical, enzymatic, or chemical methods (Hollants et al., 2010). For delicate organisms like algae, sterilization must be carefully adapted to their specific characteristics (Schulz and Boyle, 2005). Typically, this involves rinsing with sterile water, followed by treatment with 70% ethanol and sodium hypochlorite (1–4%), and finally rinsing with sterile distilled water to eliminate residual NaOCl (Stone et al., 2000; Strobel, 2002; Arnold et al., 2000). The appropriate concentration and duration of sterilization are determined based on the host and tissue type, with successful sterilization confirmed by the lack of microbial growth on the growth medium (Schulz and Boyle, 2005). Post-sterilization, samples are aseptically dissected and transferred to culture media, often supplemented with antibiotics like chloramphenicol, streptomycin, tetracycline, or penicillin to curb bacterial contamination (Tupac Otero et al., 2002). After incubation at 26 °C, fungal hyphal tips are isolated for subculturing, and the strains are archived. Through repeated transfers, endophytes are purified from the interior tissues (Suryanarayanan et al., 2003). Figure 3 provides a visual overview of the marine endophyte isolation process and metabolite profiling.

### 5.2 Strain improvement of marine endophytic fungi

The inherent activity of marine endophytic fungal strains found in nature is often insufficient for industrial-scale production of secondary metabolites. To bridge this gap, strain improvement techniques are imperative to enhance their productivity. With the advancement of biotechnology, methods such as mutagenesis and genetic engineering have become central to boosting the metabolite yield of these fungi (Demain and Adrio, 2008; Kong et al., 2022; Kong et al., 2021). Mutagenesis, both physical and chemical, is a traditional approach to induce genetic changes in microorganisms. Physical mutagens like ultraviolet radiation and chemical agents including alkylating compounds are used to increase the genetic variation, which can lead to strains with improved metabolite production (Shima et al., 1996; Hu et al., 2002). Resistance screening, leveraging antibiotic resistance as a selection tool, is a straightforward and effective method for isolating strains with desirable traits (Partridge et al., 2018). However, mutagenesis breeding suffers from inherent limitations including uncontrollable mutation sites and phenotypic instability. As evidenced by Khoshbakht et al.’s study, only 5 novel chalaniline derivatives were successfully generated from 23 precursor modifications (Khoshbakht et al., 2021). The low positive mutation rate significantly escalates both time investment and operational costs.

Protoplast fusion, a technique that merges cells by fusing their protoplasts, has been instrumental in developing high-yielding strains. This method was employed to develop a recombinant strain of *Streptomyces pristinaespiralis* with enhanced pristinamycin production capability. Through four rounds of protoplast fusion and screening, the obtained recombinant strain G4-17 achieved a pristinamycin yield of 0.89 g/L, representing a 145.9% increase compared to the original strain, while demonstrating excellent genetic stability (Xu et al., 2008). These results confirm the potential of protoplast fusion methodology for microbial strain improvement. While protoplast fusion offers significant potential, it faces considerable challenges in overcoming species barriers and may compromise the integrity of secondary metabolic gene clusters.

Metabolic engineering offers a targeted strategy for enhancing the biosynthesis of specific metabolites. By understanding the metabolic pathways and identifying rate-limiting steps, metabolic engineers can redirect the flow of metabolites towards the desired products (Kong et al., 2021). This can be achieved by overexpressing key genes or introducing synthetic gene clusters into the fungi. The endophytic paclitaxel-producing fungus *Ozonium* sp. EFY-21 represents a successful case of metabolic engineering for enhancing the production of high-value compounds (Wei et al., 2010). Studies demonstrated that by introducing the rate-limiting enzyme gene *taxadiene synthase (ts)* to modify the paclitaxel biosynthetic pathway, the paclitaxel yield in engineered transformant T4 significantly increased from 87.4 ± 6.3 μg/L in the wild-type strain to 417.1 ± 22.3 μg/L, achieving a 3.77-fold enhancement (Wei et al., 2012). However, metabolic engineering faces limitations in pathway elucidation, with the vast majority of biosynthetic gene clusters (BGCs) in marine fungi remaining functionally uncharacterized (Kumar et al., 2018). Moreover, heterologous expression may reduce enzymatic activity in certain cases, significantly diminishing the synthesis yield of target metabolites.

In summary, technological integration represents a breakthrough strategy. The synergistic combination of mutagenesis, protoplast fusion, and metabolic engineering significantly enhances the robustness of industrial microbial strains, thereby enabling sustainable and efficient production of high-value secondary metabolites. This approach is crucial for meeting industrial-scale metabolite production demands and facilitates the discovery of novel compounds with therapeutic potential.

### 5.3 Metabolic pathway engineering strategies for marine endophytic fungi

Beyond strain optimization, fermentation conditions significantly impact the production of target metabolites and their precursors in marine endophytic fungi. A well-designed fermentation process is essential to realize the full potential of engineered strains for natural product synthesis (Lau et al., 2002; Singh et al., 2017). Target product yields can be enhanced through precursor feeding, fermentation medium optimization, and the strategic use of inducers and adsorbent resins (Parekh et al., 2000). Fermentation conditions, including medium composition, pH, temperature, and stirring speed, are critical for improving secondary metabolite yields. The OSMAC (One Strain Many Compounds) strategy, pioneered by Bode et al. in 2002(Bode et al., 2002), systematically modulates culture parameters (e.g., medium composition, salinity, physical state) to activate silent biosynthetic gene clusters, thereby greatly expanding the metabolic diversity of a single strain (Wang et al., 2014). For instance, the endophytic fungus *Hypomontagnella monticulosa* cultivated in Wickerham medium produced 23 metabolites including antibacterial and anticancer briarane-type diterpenes (Lutfia et al., 2024). Comparative cultivation of *Fucus vesiculosus* symbionts in liquid vs. solid media resulted in 40% condition-exclusive metabolic nodes, with specific media inducing anticancer activity (Fan et al., 2019). Addition of NaI to rice medium triggered the production of unprecedented sulfur-containing alkaloids (aplospojaveedins A–C) in *Aplosporella javeedii* (Gao et al., 2024). These cases demonstrate OSMAC’s power to unlock novel chemical scaffolds and diversify metabolite profiles, proving essential for discovering antimicrobial and anticancer lead compounds. Inducer selection and timing are crucial for maximizing the yield of microbial secondary metabolites. By considering the physiological state and growth capacity of engineered strains, the appropriate induction conditions can be determined to enhance the expression of exogenous pathway proteins and target product yield (Sassi et al., 2016). Precursors feeding is another effective strategy, as demonstrated by the significant increase in argenocarcin production through proline and glucose supplementation (Dhakal et al., 2016).

*In situ* product removal (ISPR) is a valuable technique for managing self-toxic metabolites, ensuring high product levels and preventing their detrimental effects on microbial growth (Singh et al., 2010; Schügerl and Hubbuch, 2005). Solid adsorbents, such as polymer resins, are preferred over liquid solvents due to their lower toxicity risk and are widely applicable in endophytic fungal fermentation (Xu et al., 2009). The use of inert solid carriers has also been shown to enhance metabolite production and discovery (Bigelis et al., 2006). Environmental stimuli in liquid media can influence fungal development and metabolism, affecting metabolite production. This strategy has been effectively utilized in the fermentation of *Phomopsis* sp., where the use of adsorbent materials increased mycoepoxydiene production (Thammajaruk et al., 2011). These metabolic engineering strategies are pivotal for planning and executing the efficient production of secondary metabolites in marine endophytic fungi.

In conclusion, marine endophytic fungi represent not only integral components supporting the health and function of marine ecosystems but also constitute a treasure trove of high-value bioactive substances due to their unique metabolic capabilities and adaptation to diverse ecological niches (El-Bondkly et al., 2021; Sahoo et al., 2021; Tan et al., 2023). Their ability to produce a wide array of specialized metabolites in response to environmental stressors positions them as a promising and sustainable source for discovering new drugs and advancing biotechnological applications (Sahoo et al., 2021; Tan et al., 2023). As essential synthesis factories for secondary metabolites, they offer a scalable solution to current bottlenecks in drug discovery and development within the marine biotechnology sector, underscoring the critical importance of continued research and exploration in this field.

## 6 Discussion

Marine endophytic fungi inhabit diverse marine ecosystems, constituting an underexplored reservoir of biodiversity. These symbiotic microorganisms serve as crucial sources of structurally diverse and biologically significant secondary metabolites (e.g., anticancer, antimicrobial, and antioxidant compounds), further highlighting their potential as a valuable resource for biotechnological innovation (El-Bondkly et al., 2021; Wang et al., 2025). However, industrial applications currently face bottlenecks such as genomic instability and metabolic yield fluctuations under pure culture conditions (Shabana et al., 2021). Prevailing research predominantly focuses on strain isolation and preliminary activity screening, suffering from methodological homogeneity and insufficient quantitative production data, which severely hinders the translation from basic research to industrial applications.

To achieve efficient resource utilization, a transition from the conventional “species–compound–activity” model to a resource-driven research paradigm is imperative. This paradigm emphasizes dual-track advancement through strain improvement and process optimization: Strain enhancement: Integrating mutagenesis, protoplast fusion, and metabolic engineering to boost strain stability and biosynthetic efficiency; Process innovation: Implementing dynamic fermentation control, precision addition of elicitors/precursors, and targeted adsorption techniques to enhance metabolite production. For instance, our earlier work significantly increased the production of L-piperazic acid and putrescine in *Aureobasidium melanogenum* by employing metabolic engineering and optimized culture conditions, thereby validating the pivotal role of process engineering (Kong et al., 2022; Kong et al., 2021).

The integration of synthetic biology and systems biology heralds a transformative era in gene cluster mining. CRISPR-Cas9-mediated activation of silent biosynthetic gene clusters (BGCs) will enable the discovery of novel molecular scaffolds (e.g., isocoumarins, aminofulvenes). For instance, CRISPR-Cas9-mediated disruption of the *Fusarium graminearum*C16 BGC (targeting polyketide synthase PKS15 and terpene synthase TS genes) confirmed its products as decalin-containing diterpenoid pyrones FDDP-D and FDDP-E (Noor et al., 2020). Future efforts should combine bioinformatics-driven BGC prediction (e.g., antiSMASH analysis) with optimized heterologous expression platforms (e.g., yeast artificial chromosome systems) to reconstruct complex pathways directionally.

Advancements in PDB technology represent a pivotal breakthrough. Khoshbakht et al. successfully generated five novel chalaniline derivatives via 23 precursor modifications, demonstrating the enzymatic flexibility of fungal systems (Khoshbakht et al., 2021). Future strategies should integrate machine learning-assisted precursor design with enzyme engineering (e.g., P450 enzyme specificity modulation) for customized production of bioactive molecules. Concurrently, developing bionic fermentation systems (e.g., algal-fungal co-culture mimicking host microenvironments) could resolve metabolic instability in pure cultures, facilitating scaled-up production of algal-derived metabolites.

To establish a fully-integrated development system that bridges the pathway from strain to product, a “Strain-Process-Product” trinity framework serves as the ultimate solution. This includes: 1. Strain improvement: Integrating mutagenesis and genomic reprogramming to enhance the robustness of industrial strains; 2. Intelligent fermentation: Coupling real-time metabolic sensing with adaptive control (e.g., gradient elicitor release technology) to increase product titers; 3. Green separation: Developing biomimetic adsorption materials (e.g., functionalized XAD-7 resins) to reduce downstream purification costs. Through interdisciplinary technological integration, marine endophytic fungi are poised to become highly efficient “cell factories,” providing a sustainable repository of high-value natural products for drug development.

## 7 Future perspective

To fully unlock the potential of marine endophytic fungi as sustainable sources of high-value natural products, future research must adopt an integrated and interdisciplinary strategy. Moving beyond traditional isolation and screening approaches, efforts should prioritize the development of robust, industrially applicable systems through synergistic advances in strain engineering, process control, and pathway discovery. Key directions will include: leveraging CRISPR-Cas9 and synthetic biology tools to activate silent biosynthetic gene clusters and enable heterologous production of novel compounds; employing machine learning and enzyme engineering to optimize precursor-directed biosynthesis and metabolic flux; and designing bionic co-culture systems to mimic native host microenvironments and stabilize metabolic output. Ultimately, the implementation of a holistic “Strain–Process–Product” framework—combining genetically enhanced strains, intelligently controlled fermentation, and eco-friendly downstream purification—will transform these fungi into efficient cell factories, bridging the gap between laboratory discovery and industrial-scale production of pharmaceuticals and other bioactive compounds.
