# Correction: Marine endophytes: biosynthetic engines for novel bioactive metabolites

**DOI:** 10.3389/fmicb.2025.1721084

**Published:** 2025-10-24

**Authors:** Cun-Cui Kong, Jia-Yi Wang, Bo-Hao Shan, Hong-Xia Zhang, Song Qin, Cheng-Gang Ren

**Affiliations:** ^1^College of Horticulture, Ludong University, Yantai, Shandong Province, China; ^2^Key Laboratory of Coastal Biology and Biological Resources Utilization, Yantai Institute of Coastal Zone Research Chinese Academy of Sciences, Yantai, China

**Keywords:** marine endophytic fungi, bioactive metabolites, strain improvement, fermentation optimization, biosynthetic potential

In the published article, the author, Cun-Cui Kong was erroneously assigned as the corresponding author. The correct corresponding author is Cheng-Gang Ren.

In the published article, the author, Hong-Xia Zhang was incorrectly assigned to affiliation 2, Key Laboratory of Coastal Biology and Biological Resources Utilization, Yantai Institute of Coastal Zone Research Chinese Academy of Sciences, Yantai, China. The author is only affiliated to affiliation 1, College of Horticulture, Ludong University, Yantai, Shandong Province, China.

In the published article, the names of the following authors were incorrectly written as “Jiayi Wang” and “Bohao Shan”. The correct spellings of their names are “Jia-Yi Wang” and “Bo-Hao Shan”.

The original version of this article has been updated.

